# Differential association of abdominal, liver, and epicardial adiposity with anthropometry, diabetes, and cardiac remodeling in Asians

**DOI:** 10.3389/fendo.2024.1439691

**Published:** 2024-08-23

**Authors:** Vivian Lee, Yiying Han, Desiree-Faye Toh, Jennifer A. Bryant, Redha Boubertakh, Thu-Thao Le, Calvin W. L. Chin

**Affiliations:** ^1^ National Heart Research Institute Singapore (NHRIS), National Heart Centre Singapore, Singapore, Singapore; ^2^ Department of Cardiology, National Heart Centre Singapore, Singapore, Singapore; ^3^ Cardiovascular Academic Clinical Program (ACP), Duke-National University of Singapore (Duke-NUS) Medical School, Singapore, Singapore

**Keywords:** cardiometabolic disease, fat distribution, visceral adiposity, diabetes, anthropometric indices, epicardial fat, cardiac remodeling, magnetic resonance imaging

## Abstract

**Background:**

Heterogenous deposition and homeostasis roles of physiologic and ectopic adipose tissues underscore the impact of fat compartmentalization on cardiometabolic risk. We aimed to characterize the distribution of abdominal visceral adipose tissue (VAT), subcutaneous adipose tissue (SAT), epicardial adipose tissue (EAT), and liver fat on magnetic resonance imaging (MRI), and evaluate their associations with anthropometric indices and adverse cardiac remodeling.

**Methods:**

In this cross-sectional observational study, 149 Asian adults (57.0 ± 12.8 years; 65% males) with at least one cardiometabolic risk factor underwent multiparametric fat and cardiovascular MRI. Anthropometric indices included body mass index (BMI), waist circumference (WC), waist-hip ratio (WHR), and bioimpedance body fat mass (BFM). Associations between fat depots and anthropometric measures as well as cardiac remodeling features were examined as a single cohort and stratified by type 2 diabetes mellitus (T2DM) status.

**Results:**

VAT and SAT had opposing associations with liver fat and EAT. Therefore the VAT/SAT ratio was explored as an integrated marker of visceral adiposity. VAT/SAT was positively associated with EAT (β=0.35, P<0.001) and liver fat (β=0.32, P=0.003) independent of confounders. Of the anthropometric measurements assessed, only WHR was independently associated with VAT/SAT (β=0.17, P=0.021). Individuals with T2DM had higher VAT and lower SAT compared to those without T2DM, translating to a significantly higher VAT/SAT ratio. EAT volume was independently associated with adverse features of cardiac remodeling: increased left ventricular (LV) mass (β=0.24, P=0.005), larger myocyte volume (β=0.26, P=0.001), increased myocardial fibrosis (β=0.19, P=0.023), higher concentricity (β=0.18, P=0.035), and elevated wall stress (β=−0.18, P=0.023).

**Conclusion:**

Multiparametric MRI revealed abdominal VAT and SAT have differential associations with anthropometric indices and ectopic fats in a single cohort of Asians at risk of cardiometabolic disease. People with T2DM have expanded VAT and diminished SAT, endorsing the VAT/SAT ratio beyond usual anthropometric measurements as a marker for multiorgan visceral fat composition. Among the fat depots examined, EAT is uniquely associated with adverse cardiac remodeling, suggesting its distinctive cardiometabolic properties and implications.

## 1 Introduction

Disequilibrium or dysfunctional adipose tissue leads to obesity and metabolic disorders which in turn are risk factors for cardiovascular complications, described as cardiometabolic disease (1). As major fat depots, visceral adipose tissue (VAT) and subcutaneous adipose tissue (SAT) are believed to have distinct metabolic roles. Ectopically, abnormal expansion of epicardial adipose tissue (EAT) is associated with coronary artery disease (CAD), heart failure (HF), and atrial fibrillation (AF) (2) whereas excessive liver fat accompanying metabolic dysregulation in metabolic dysfunction-associated fatty liver disease (MAFLD) is implicated in elevated cardiometabolic risk (3).

The metabolic heterogeneity of adipose tissues in type, size, function, and distribution as well as their modifiable potential indicate the need for fat phenotyping, enabled by advances in imaging-based quantification. However, the utility of conventional anthropometric indices for accurate assessment of adiposity is often challenged by the inability to account for the anatomical composition of fat and the multifactorial variation of body habitus. Alternative measures have been proposed but have yet to become mainstays of obesity diagnosis and stratification.

Current data suggest that Asians have a higher propensity for visceral fat storage and develop metabolic syndrome at a lower body mass index (BMI) (4). We aim to characterize the distribution of abdominal VAT and SAT, liver fat, and EAT in Asians at risk of cardiometabolic disease using multiparametric MRI. Associations of fat composition with anthropometric indices and features of cardiac remodeling were examined. We hypothesized heterogeneous associations among these fat depots with anthropometric indices and cardiac remodeling characteristics, which are distinguished by glycemic status.

## 2 Materials and methods

### 2.1 Study population

This observational study consisted of participants selected from the National Heart Centre Singapore Biobank, who had at least one of the following cardiometabolic risk factors: hypertension, type 2 diabetes mellitus (T2DM), hyperlipidemia, fatty liver, increased BMI, and abdominal obesity. Individuals with inherited cardiomyopathies (hypertrophic, dilated and infiltrative cardiomyopathies) were excluded from the study.

Parameters evaluated included anthropometric measurements, body fat mass by bioimpedance analysis, MRI-quantified adipose tissue (abdominal VAT and SAT areas, liver fat fraction, EAT volume), and cardiac metrics (mass, volumes, myocardial fibrosis markers, wall stress) using cardiovascular magnetic resonance (CMR).

Ethics approval was granted by the SingHealth Biobank Research Scientific Advisory Committee (SBRSA 2019/001). The study was performed in accordance with ethical principles that have their origin in the Declaration of Helsinki. All study participants provided written informed consent.

### 2.2 Anthropometric indices and bioimpedance body fat analysis

Anthropometric measurements acquired with standard methods included BMI, waist circumference (WC), and waist-hip ratio (WHR). BMI was calculated as weight (kg)/height (m)^2^. WC was measured at just above the navel. Hip circumference was taken at the widest portion of the hip area. The ratio between waist and hip circumferences was calculated as WHR. All measurements were taken in a standing position. Bioimpedance analysis (InBody, Cerritos, California, USA) was used to measure body fat mass (BFM), calculated as the difference between total body mass and fat-free mass that was made up of water, protein, and minerals.

Local BMI thresholds guided by the recommendations made by the WHO Expert Consultation Panel were used to define normal (<23.0 kg/m^2^), overweight (23.0-30.0 kg/m^2^), and obese (>30.0 kg/m^2^) (5). Abdominal obesity was defined as WHR >0.90 and >0.85 or WC >90 cm and >80 cm for males and females, respectively (6).

### 2.3 Abdominal and cardiac MRI acquisition

MRI was performed for all participants using the 1.5T Siemens Aera (Siemens Healthineers, Erlangen, Germany). Abdominal VAT and SAT were examined from a series of contiguous cross-sectional abdominal water- and fat-separated images obtained from the two-point Dixon method (TEs: 2.39 and 4.77 ms; TR: 6.5 ms; flip angle: 10 degrees; matrix size: 260 × 320 mm^2^; FOV: 325-366 × 400-450 mm^2^; slice thickness: 4 mm, slice gap: 0.8 mm) (7). Liver proton density fat fraction (PDFF) was acquired according to Liver*MultiScan*-Iterative Decomposition of water and fat with the Echo Asymmetry and Least Squares estimation method (*LMS IDEAL*; Perspectum Ltd, Oxford, London), which has been implemented across MRI platforms (8). The sequence parameters were as follows: TEs: 1.30, 3.30, 5,30, 7.30, 9.30, and 11.30 ms; TR: 14 ms; flip angle: 5 degrees; matrix size: 232 × 256 mm^2^; FOV: 398 × 440 mm^2^; slice thickness:10 mm; number of slices: 5; slice gap: 5 mm.

For CMR, balanced steady-state free precession cine images were acquired in the long-axis 2, 3, 4 chamber views, and short-axis view extending from the mitral valve annulus to the apex (acquired voxel size: 1.6 × 1.3 × 8.0 mm^3^; slice gap: 2mm; 30 phases per cardiac cycle). Late gadolinium enhancement (LGE) imaging denoting replacement myocardial fibrosis was performed 8 minutes after 0.1 mmol/kg of gadobutrol administration (Gadovist; Bayer Pharma AG, Germany). A breath-held inversion-recovery fast gradient echo sequence was used, and the inversion time was optimized to achieve appropriate nulling of the myocardium. The native and 15-minute postcontrast myocardial T1 maps were acquired with a modified Look-Locker inversion-recovery sequence, applying a heartbeat acquisition scheme of 5(3)3 and 4(1)3(1)2, respectively.

### 2.4 MRI-based fat and cardiac analysis

De-identified abdominal and cardiac images were analyzed at Perspectum and the National Heart Research Institute (NHRIS) Core Laboratory, respectively, by trained individuals who were blinded to clinical data. Abdominal VAT, SAT, and liver PDFF were analyzed by Perspectum’s expertly trained image analysts who were blinded to the clinical data.

**Figure 1:**
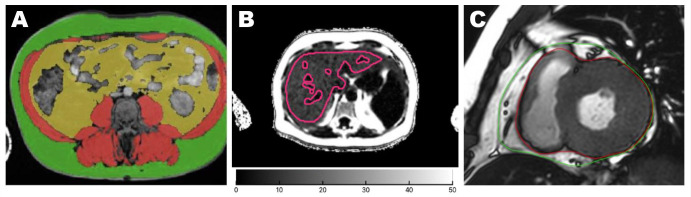
Quantification of adipose tissue from MRI axial images. Areas of abdominal adipose tissues were acquired at the vertebral L3 level. Green: SAT; yellow: VAT **(A)**. Liver PDFF was quantified from the whole liver region of interest marked in red lines on a parametric map, avoiding image artifacts and major vessels **(B)**. EAT was segmented between the epicardium (red line) and the pericardium (green line) on both ventricles at end-systole from the basal to apical short-axis cines. The paracardial fat outside the pericardium was not included in the EAT contour **(C)**. EAT, epicardial adipose tissue; SAT, subcutaneous adipose tissue; VAT, visceral adipose tissue.

Cross-sectional areas of VAT and SAT were segmented from the abdominal Dixon MRI image at the L3 vertebral level using ITK-SNAP software version 3.8 (PICLS, University of Pennsylvania, USA) (**Figure 1A**). This single-slice approach of quantifying VAT and SAT has been shown to correlate strongly with total SAT and VAT volumes (r>0.9 in men and women) (9, 10). Liver fat was quantified as PDFF, expressed as a percentage, and computed as fat/(fat+water) based on MRI-visible fat and water signals in the regions of interest placed on the PDFF parametric map, avoiding image artifacts and vessels (11, 12) (**Figure 1B**). Fatty liver was defined as PDFF >5.6% (13, 14). Liver*MultiScan* reports IDEAL-acquired PDFF with an accuracy within 3% of lab-analyzed fat samples (15).

EAT volume on the left and right ventricles was quantified at the end systole on short-axis cines extending from the mitral valve annulus to the apex using CVI42 (Circle Cardiovascular Imaging, Calgary, Canada). The bright layer between the myo-epicardial border and the pericardium constituted the EAT. EAT was carefully delineated along the pericardium to exclude the paracardial fat which sits outside the margin of the pericardium (**Figure 1C**).

LV mass and cardiac volumes were analyzed according to standardized protocols (16). LV concentricity was defined as the ratio of LV mass over end-diastolic volume (EDV) (17). The Remodeling Index (RI) is a surrogate marker of global myocardial wall stress, calculated as $RI=\sqrt[{3}]{EDV}/t$ , where EDV is the LV end-diastolic volume (mL) and *t* is the maximal wall thickness (cm) across the 16 myocardial segments (18). A lower RI denotes increased global myocardial wall stress and predicts worse cardiovascular outcomes in individuals with hypertension (19).

Interstitial volume (mL), as a measure of diffuse interstitial myocardial fibrosis, was calculated as the extracellular volume (ECV) fraction × myocardial volume (mL), where extracellular volume (ECV) was quantified from native and post-contrast T1 maps, and myocardial volume was calculated by dividing the myocardial mass by the specific gravity of the myocardium (1.05 g/mL). Myocyte volume (mL) = myocardial volume – interstitial volume (20).

### 2.5 Statistical analysis

The normality of data distribution was assessed with the Shapiro-Wilk test. Continuous variables were presented as mean ± standard deviation (SD) if normally distributed or median (interquartile range) if otherwise. Categorical variables were expressed as frequency (percentage) and were analyzed using the χ^2^ test.

Depending on the continuous or categorical nature of the dependent variable, multivariable linear or logistic regression analyses with adjustment for potential confounders were performed to evaluate the associations between (i) anthropometric indices and adipose tissues; (ii) abdominal, epicardial, and liver adipose tissues; and (iii) adipose tissues and cardiac remodeling markers. Clinically important potential confounders, including age, sex, ethnicity, BMI, systolic blood pressure (SBP), hyperlipidemia, and T2DM status, were adjusted for where applicable.

Mean differences in adipose tissues between categorical groups (WHR categories, BMI categories, T2DM status) were adjusted for confounders and compared using a one-way analysis of covariance (ANCOVA). *Post hoc* Bonferroni was performed for pairwise comparison between BMI categories (normal, overweight, and obese). Adjusted mean differences with a 95% confidence interval (CI) were reported. We compared the ability of VAT, SAT, and VAT/SAT ratio to differentiate individuals with and without T2DM using the area under the receiver operating characteristic curve (AUC).

Statistical significance was defined as P<0.05. Statistical analyses were performed using IBM SPSS Statistics Version 26 (IBM Corp, Armonk, NY, USA) and GraphPad Prism Version 7.05 (GraphPad Software, Inc, La Jolla, CA, USA).

## 3 Results

A total of 149 participants (57.0 ± 12.8 years old; 65% males; 83% Chinese) with cardiometabolic risk factors had MRI assessment of compartmental fat and cardiac remodeling (**Table 1**). Mean BMI was 26.9 ± 4.2 kg/m^2^ and mean WHR was 0.93 ± 0.08 (males: 0.96 ± 0.06, females: 0.87 ± 0.09; P<0.001). The prevalence of normal, overweight, and obese status defined by BMI was 22%, 58%, and 20%, respectively. These participants had other cardiometabolic risk factors including hypertension (n=120, 81%), T2DM (n=56, 38%), hyperlipidemia (n=75, 50%), and fatty liver (PDFF=6.7 [3.3-14.3] %). A greater proportion of T2DM participants were men (n=43, 77%; P=0.020 for sex difference) but the proportion of hyperlipidemia and fatty liver was not significantly different between sexes (P=0.275 and P=0.913, respectively).

**Table 1:** Baseline characteristics.

Demographics and Clinical Characteristics	
Age, years	57.0 ± 12.8
Male, n (%)	97 (65.1)
Chinese, n (%)	124 (83.2)
24-hour mean SBP, mmHg	131 ± 13
24-hour mean DBP, mmHg	80 ± 11
Hypertension, n (%)	120 (80.5)
Hyperlipidemia, n (%)	75 (50.3)
T2DM, n (%)	56 (37.5)
Fatty liver, n (%)	84 (56.4)
Ischemic heart disease, n (%)	4 (2.7)
HbA1c, %^∏ ^	7.0 (6.5-7.5)

Anthropometric and Bioimpedance Indices	
Body surface area, m^2^	1.79 ± 0.21
Weight, kg	73.1 ± 14.6
Height, m	1.64 ± 0.09
BMI, kg/m^2^	26.9 ± 4.2
Waist circumference, cm	93 ± 12
Hip circumference, cm	100 ± 8
WHR	0.93 ± 0.18
Bioimpedance BFM, kg	24.4 ± 8.3
Skeletal muscle mass, kg	27.5 ± 8.7
Adipose Tissue Characteristics	
VAT area, cm^2^	170.8 ± 82.7
SAT area, cm^2^	180.5 ± 87.3
VAT/SAT ratio	1.10 ± 0.68
EAT volume, cm^3^	113.2 ± 38.5
Liver PDFF, %	6.7 (3.3-14.3)

Cardiovascular Magnetic Resonance Characteristics	
Indexed LV mass, g/m^2*^	50 ± 11
Indexed LV EDV, mL/m^2*^	70 ± 12
Indexed LV ESV, mL/m^2*^	29 ± 8
Indexed LV SV, mL/m^2*^	41 ± 7
LV EF, %	59 ± 6
LV mass/EDV ratio	0.71 ± 0.15
Indexed RV EDV, mL/m^2*^	71 ± 13
Indexed RV ESV, mL/m^2*^	30 ± 10
Indexed RV SV, mL/m^2*^	41 ± 7
RV EF, %	58 ± 9
Late gadolinium enhancement, n (%)	28 (18.8)
Extracellular volume fraction, %	25.1 ± 2.5
Indexed interstitial volume, mL/m^2*^	11.9 ± 2.6
Indexed myocyte volume, mL/m^2*^	35.6 ± 7.9
Remodeling index	5.9 ± 1.1
Global longitudinal strain, %	-16.6 ± 2.3
Global circumferential strain, %	-19.4 ± 2.6
Global radial strain, %	33.9 ± 7.7

^∏ ^Only in individuals with T2DM. ^*^Indexed to body surface area calculated using the DuBois formula = 0.007184 × height (m)^0.725^ × weight (kg)^0.425^.

BFM, body fat mass; DBP, diastolic blood pressure; EDV, end-diastolic volume; EF, ejection fraction; ESV, end-systolic volume; LA, left atrial; LV, left ventricular; PDFF, proton density fat fraction; RA; right atrial; RV, right ventricular; SAT, subcutaneous adipose tissue; SBP, systolic blood pressure; SV, stroke volume; T2DM, type 2 diabetes mellitus; VAT, visceral adipose tissue; WHR, waist-hip ratio.

### 3.1 Association between anthropometric indices and fat depots

All anthropometric measurements were independently associated with VAT and SAT, with modest differences in the strength of associations: WHR was associated more strongly with VAT, whereas WC, BMI, and BFM were associated more with SAT (**Table 2**).

**Table 2:** Multivariable linear regression demonstrating independent associations between anthropometric indices and abdominal fat.

	VAT, cm^2^	SAT, cm^2^	VAT/SAT ratio
WC, cm	0.75, P<0.001	0.81, P<0.001	0.071, P=0.311
WHR	0.53, P<0.001	0.39, P<0.001	0.172, P=0.021
BMI, kg/m^2^	0.61, P<0.001	0.78, P<0.001	0.007, P=0.909
BFM, kg	0.62, P<0.001	0.78, P<0.001	0.045, P=0.470

All analyses were adjusted for age, sex, ethnicity, SBP, hyperlipidemia, and T2DM. Data presented as standardized β coefficients and corresponding P values. BFM, body fat mass on bioimpedance; BMI, body mass index; WC, waist circumference; WHR, waist hip ratio; SAT, subcutaneous adipose tissue; VAT, visceral adipose tissue.

**Figure 2:**
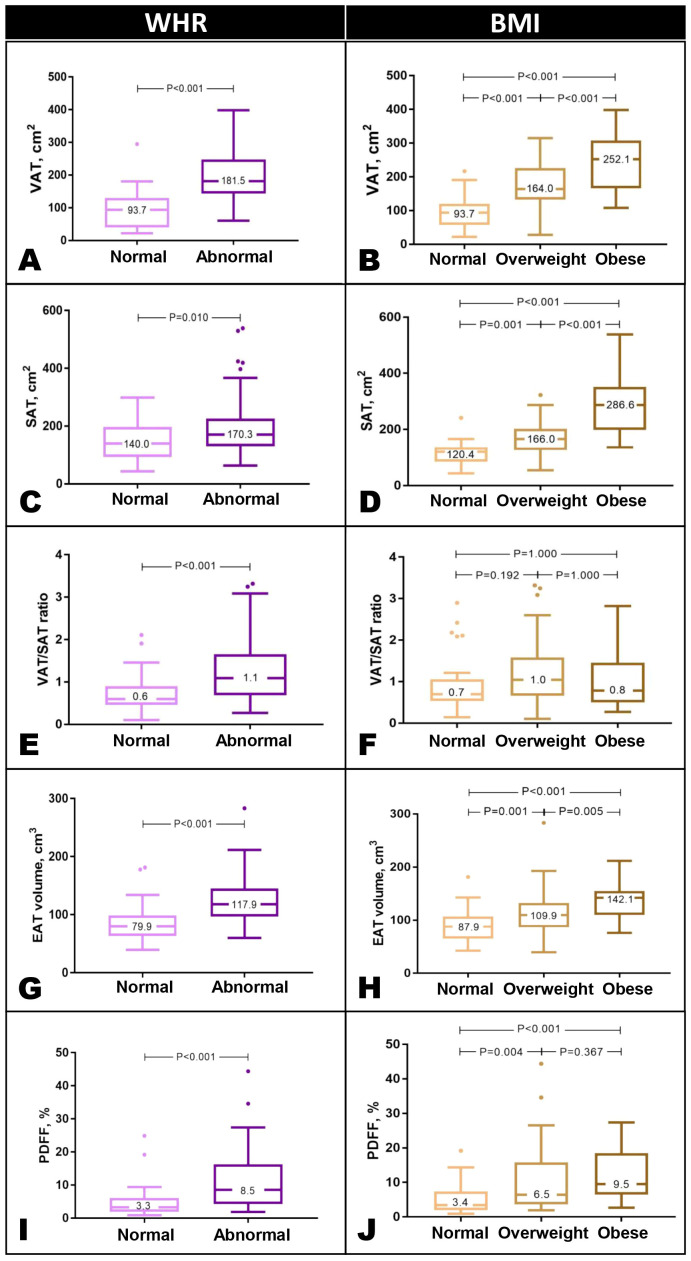
Abdominal VAT and SAT were significantly higher in individuals with abnormal WHR and across BMI categories **(A–D)**. VAT/SAT ratio was significantly increased in individuals with elevated WHR but not across BMI categories **(E, F)**. EAT was increased in those with abnormal WHR and across BMI categories **(G, H)**. Liver PDFF was greater in those with abnormal WHR. Across BMI categories, PDFF was higher than normal but not significantly different between overweight and obese individuals **(G–J)**. Results are presented in Tukey box and whisker plots. WHR thresholds: males, 0.90, females, 0.85. Asian BMI thresholds: normal <23 kg/m^2^, overweight 23-30 kg/m^2^, obese >30 kg/m^2^. BMI, body mass index; SAT, subcutaneous adipose tissue; VAT, visceral adipose tissue; WHR, waist-hip ratio.

VAT and SAT were significantly higher in individuals with abnormal WHR and BMI (**Figures 2A–D**). We explored the VAT/SAT ratio as an integrated variable to examine the relative distribution of VAT and SAT by anthropometry. VAT/SAT ratio was strongly associated with male sex (β=0.27, P<0.001), increasing age (β=0.35, P<0.001), and T2DM status (OR=4.071, P=0.001; **Supplementary Table S1**). Of all the anthropometric measures assessed, only WHR was independently associated with VAT/SAT ratio (β=0.17, P=0.021).

VAT/SAT ratio was significantly increased in those with abnormal WHR but not across BMI categories (**Figures 2E, F**). EAT volume was greater with abnormal WHR, and across BMI (**Figures 2G, H**); liver PDFF was increased in those with abnormal WHR, but not significantly different between overweight and obese individuals (**Figures 2I, J**). All findings were adjusted for age, sex, ethnicity, SBP, hyperlipidemia, and T2DM (**Supplementary Tables S2**, **S3**).

### 3.2 Association between fat depots

**Figure 3:**
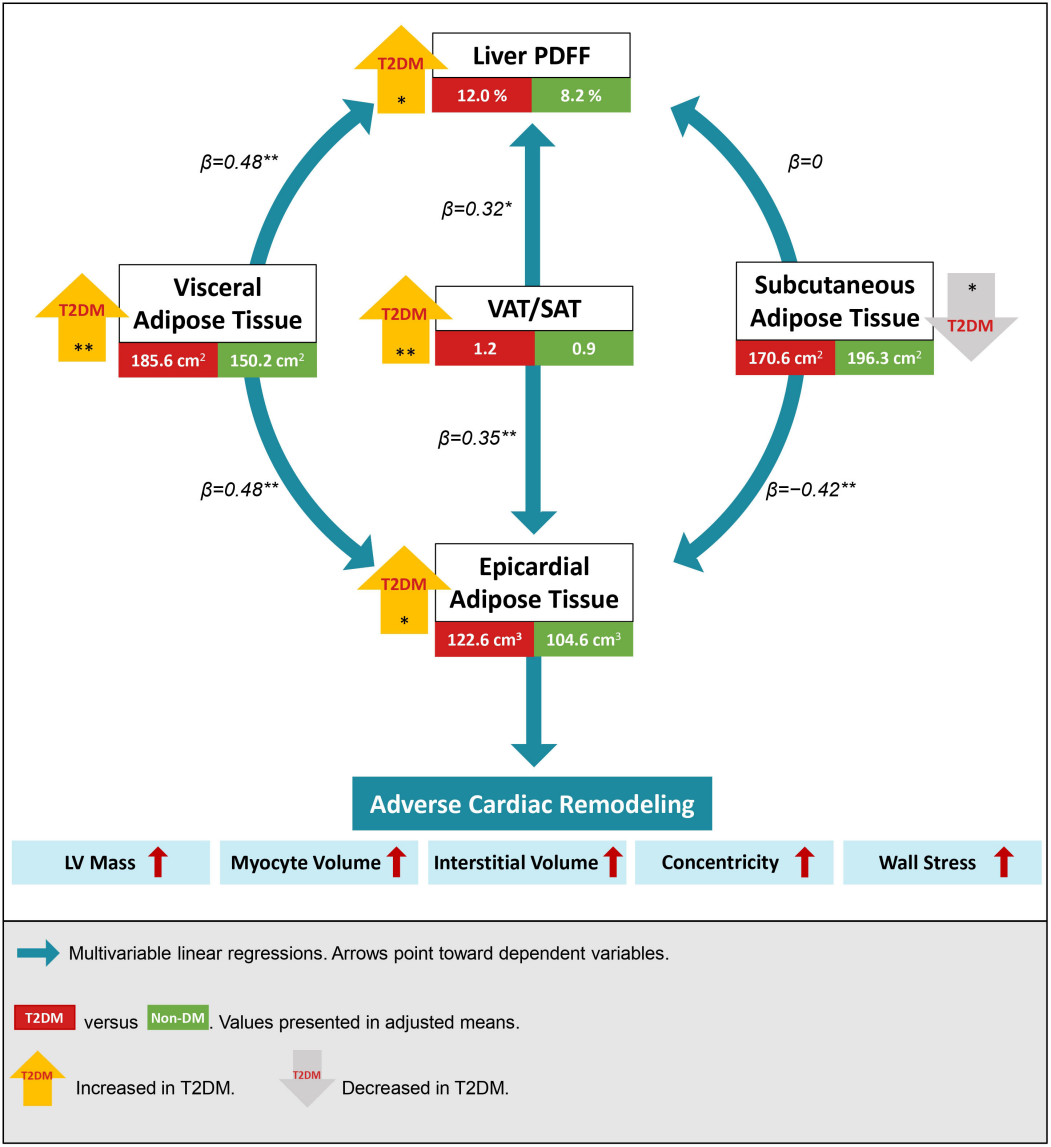
Visceral and subcutaneous adipose tissue (VAT and SAT) had inverse associations with epicardial adipose tissue (EAT) and liver proton density fat fraction (PDFF). An increase in the integrated VAT/SAT index was associated with an increase in both EAT and liver PDFF. Individuals with type 2 diabetes mellitus (T2DM) had increased visceral and ectopic fat depots. Regardless of diabetes status, adverse cardiac remodeling was observed with the accumulation of ectopic EAT. Multivariable regressions were adjusted for age, sex, ethnicity, systolic blood pressure, hyperlipidemia, body mass index, and T2DM. Mean differences in fat depots between T2DM and non-DM were analyzed using one-way ANCOVA adjusted for all confounders except T2DM status. *P<0.05; **P<0.001.

Abdominal VAT and SAT were weakly correlated with each other (r=0.19, P=0.024) and had opposing associations with EAT and liver PDFF. While an increase in VAT was associated with an increase in EAT (β=0.48; P<0.001), an increase in SAT was associated with a smaller EAT volume (β=−0.42; P=0.001). Similarly, greater VAT area was associated with higher liver PDFF (β=0.48; P<0.001) but no association was observed between SAT and liver PDFF (β=0; P=0.999) (**Figure 3**).

Using the VAT/SAT ratio to examine the contrasting effects between VAT and SAT, we observed that an increase in VAT/SAT ratio was significantly associated with an increase in both EAT (β=0.35, P<0.001) and liver PDFF (β=0.32, P=0.003) (**Figure 3**). All analyses were adjusted for age, sex, ethnicity, BMI, SBP, hyperlipidemia, and T2DM status.

Despite similar anthropometric measures (WC, WHR, BMI, and bioimpedance BFM) between individuals with and without T2DM (**Supplementary Figure S1**), individuals with T2DM had an increased propensity for visceral fat accumulation. Compared to non-diabetic participants, those with T2DM had significantly increased VAT (193.0 ± 7.2 versus 157.4 ± 5.4 cm^2^, P<0.001), less SAT (164.3 ± 7.0 versus 190.3 ± 5.3 cm^2^, P=0.006), a larger EAT volume (124.5 ± 4.5 versus 106.4 ± 3.4 cm^3^, P=0.003), and a higher liver PDFF (11.7 ± 1.1 versus 7.9 ± 0.8%, P=0.007) after adjustment for potential confounders (**Figure 3**; **Supplementary Table S4**).

**Figure 4:**
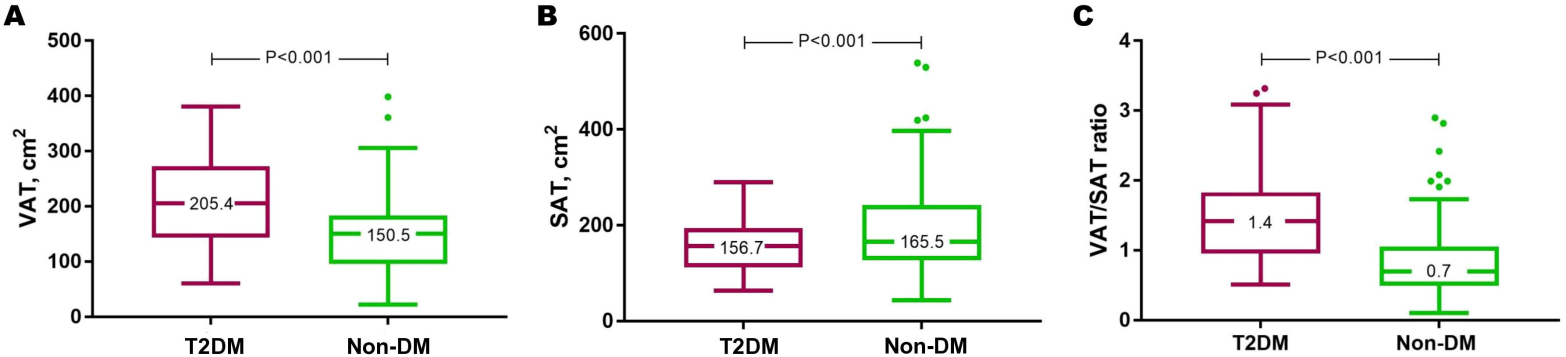
Individuals with T2DM had increased VAT **(A)**, reduced SAT **(B)**, and this translated to a higher VAT/SAT ratio **(C)**. Results are presented in Tukey box and whisker plots. DM, diabetes mellitus; SAT, subcutaneous adipose tissue; T2DM, type 2 diabetes mellitus; VAT, visceral adipose tissue.

The higher VAT and lower SAT in T2DM translated to a significantly higher VAT/SAT ratio in individuals with T2DM than those without (adjusted mean difference=0.37, 95% CI=0.18-0.56, P<0.001) (**Figure 4**; **Supplementary Table S4**). Indeed, VAT/SAT ratio demonstrated the highest discrimination for the presence of T2DM (AUC=0.79, 95% CI=0.72-0.86, P<0.001) compared to VAT (AUC=0.71, 95% CI=0.63-0.80, P<0.001) and SAT (AUC=0.62, 95% CI=0.53-0.71, P=0.019) alone (**Supplementary Table S5**).

### 3.3 Association between fat depots and CMR markers of cardiac remodeling

**Figure 5:**
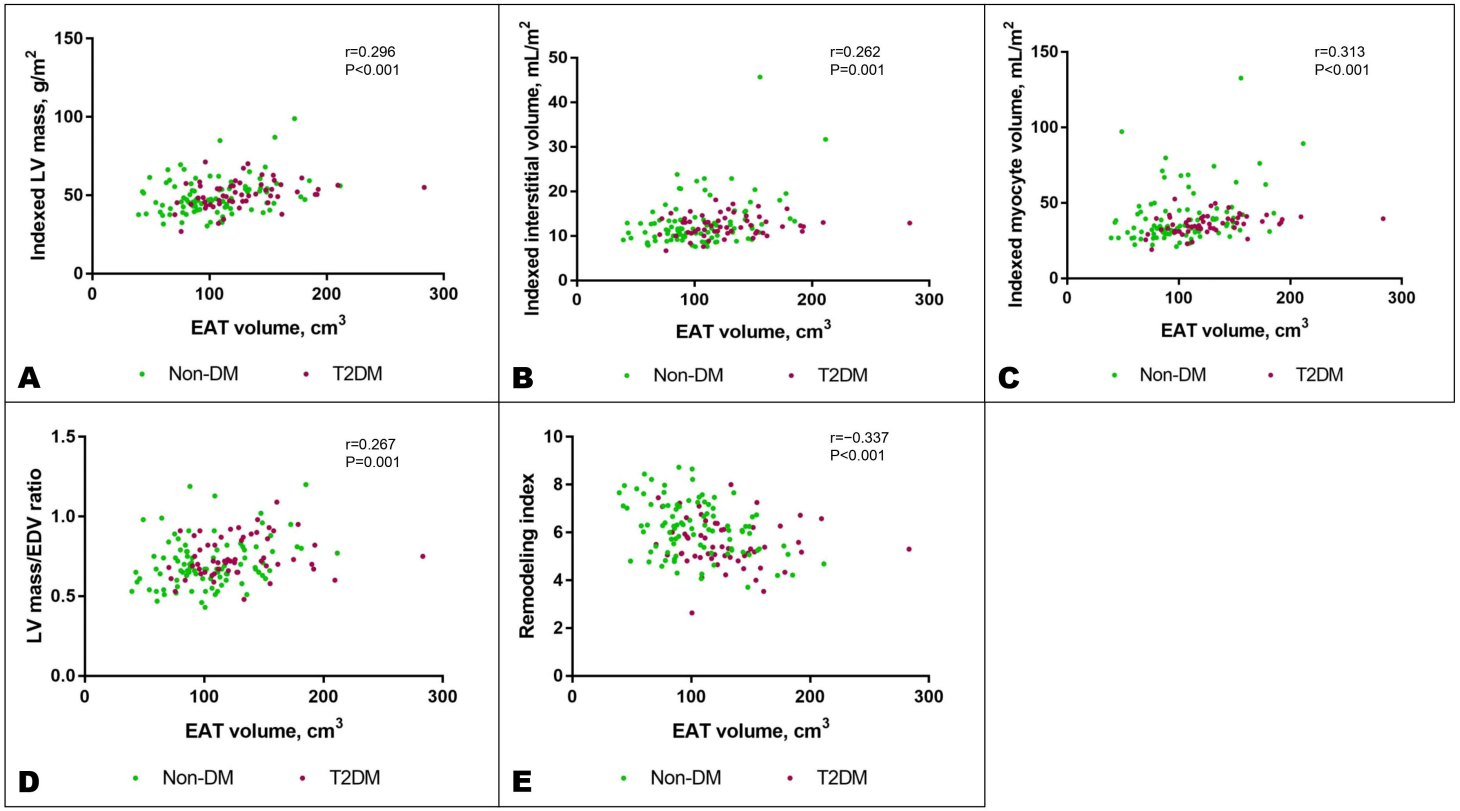
Increased EAT volume was associated with features of adverse cardiac remodeling: increased indexed LV mass **(A)**, indexed interstitial volume **(B)**, indexed myocyte volume **(C)**, LV mass/EDV ratio denoting concentricity **(D)**, and reduced remodeling index, a marker of elevated myocardial wall stress **(E)**. These associations remained significant after adjusting for potential confounders as listed in the text. DM, diabetes mellitus; EDV, end-diastolic volume; LV, left ventricular; T2DM, type 2 diabetes mellitus.

Among all the fat depots assessed in the study, EAT demonstrated consistent and independent associations with adverse features of cardiac remodeling on CMR, which remained significant after adjustment for age, sex, SBP, hyperlipidemia, and T2DM status (**Figure 3**). Specifically, an increase in EAT volume was independently associated with increased LV mass (β=0.24, P=0.004), expanded interstitial volume denoting diffuse interstitial myocardial fibrosis (β=0.19, P=0.023), larger myocyte volume (β=0.26, P=0.001), increased concentricity (LV mass/EDV ratio: β=0.18, P=0.035), and reduced RI denoting increased myocardial wall stress (β=−0.18, P=0.023). A higher VAT/SAT ratio demonstrated independent association only with increased concentricity (β=0.23, P=0.035) (**Figure 5**; **Supplementary Table S6**).

A total of 28 individuals (18.8%) had LGE on CMR (ischemic pattern consistent with infarction, n=4; non-ischemic pattern, n=24). Individuals with LGE had similar EAT volumes compared to those without (125.7 ± 33.9 versus 110.8 ± 38.8 cm^3^, P=0.064), although this should be interpreted with caution because only a small proportion of individuals had LGE.

## 4 Discussion

Our results showed that abdominal VAT and SAT were both increased in central and systemic obesity, with WHR (but not BMI classification) as a better indicator of visceral adiposity. To the best of our knowledge, this study is the first to report that VAT and SAT have inverse associations with ectopic fats and T2DM, endorsing the potential value of the VAT/SAT ratio as an indication of relative visceral adiposity. The amounts of EAT and liver fat were associated with VAT, all of which were distinctively increased in the presence of T2DM while SAT was diminished. Among the fat depots assessed, the accumulation of EAT was associated with adverse features of cardiac remodeling on CMR.

### 4.1 Relative visceral adiposity

VAT, being a metabolically active endocrine organ, confers higher cardiometabolic risks over SAT (21, 22). A cross-sectional study of 3,197 Japanese healthy adults demonstrated a greater association between VAT and metabolic diseases. SAT, by contrast, demonstrated an inverse association with impaired glucose metabolism in men and no significant association in women (23). In another study involving biopsy-proven non-alcoholic fatty liver disease (NAFLD) patients, MRI-quantified VAT was associated with insulin resistance, glucose, triglyceride, and WHR whilst SAT was negatively associated with these same indices (24). Inflammatory mediators were also differentially associated with VAT and SAT. Pro-inflammatory factors were expressed in a greater amount by VAT than SAT (25, 26). Conversely, adiponectin, which has a putative anti-inflammatory effect, was less abundant in the VAT (27). Lower levels of adiponectin were associated with a higher prevalence and increased risk of cardiovascular diseases (28, 29). These differences supported our observations of the opposite associations of VAT and SAT with T2DM, liver fat, and EAT.

Different treatments had different effects on adiposity. For instance, an improvement in glycemic profile with troglitazone therapy was accompanied by increased SAT (30) whereas glucagon-like peptide-1 receptor agonist (GLP-1RA) therapy decreased VAT and not SAT (31, 32). Furthermore, surgical removal of VAT ameliorated metabolic syndrome while the effects of SAT removal have been inconsistent, with some demonstrating an improved (33, 34) or neutral (35, 36) effect on insulin sensitivity. For these reasons, the absolute amount of either of these fat depots may not fully reflect obesity-related cardiometabolic risk. Visceral adiposity, which has an established link with insulin resistance (37, 38), is therefore an appealing option that may be represented by the VAT/SAT ratio. Our findings corroborated this postulation, demonstrating the superior ability of the VAT/SAT ratio to discriminate diabetes status over VAT and SAT alone. This integrated index, interpreted in the context of the absolute amount of SAT and VAT, will likely inform a more complete metabolic profile of an individual.

### 4.2 Anthropometric indices and fat depots

In our study, BMI correlated weakly with VAT, EAT, and liver PDFF compared with waist-derived measurements. The advantage of WHR in estimating visceral adiposity is substantiated by the ability of abnormal sex-specific WHR to indicate an increased VAT/SAT ratio. In contrast, the Asian-specific BMI classification showed no difference in the VAT/SAT ratio between normal, overweight, and obese status. BMI’s inference on obesity as a function of weight and height lacked the sensitivity to delineate body fat composition. Such inadequacy in reflecting true adiposity and the associated cardiometabolic risk has given rise to phenomena such as normal-weight obesity (39, 40) and metabolically healthy obesity (41). Despite accounting for muscle, water, and bone mass, bioimpedance BFM is a measure of generalized fat storage and does not differentiate anatomical fat distribution. The predominant localization of VAT around the intra-abdominal organs and SAT in the gluteal-femoral region (42, 43) may suggest WHR as the preferred surrogate measure for abdominal visceral adiposity.

### 4.3 Abdominal, epicardial, and liver fat depots

Parallel with predominant visceral adiposity at the abdominal level, we observed greater EAT volume and liver fat in patients with T2DM. EAT is considered the true visceral fat of the heart as it originates from the same embryonic layer as VAT and is in direct contact with the myocardium (44). Our finding is in line with this theory such that EAT was positively associated with VAT but negatively with SAT. Notably, in non-diabetic patients, although they had relatively more abdominal SAT, there was a good correlation between their VAT/SAT ratio and EAT volume, implying that EAT size could be approximated by relative abdominal visceral adiposity regardless of their diabetes status.

On a similar note, the liver PDFF was significantly higher with more abdominal VAT but not SAT. This differential association between abdominal fat with PDFF could be explained by insulin resistance exacerbated by visceral adiposity. The free fatty acids of VAT are drained by the portal vein (45) which when increased in metabolic disorders would lead to hepatic steatosis (46, 47). Whilst NAFLD increases the risk of cardiac complications (48), NAFLD patients with expanded EAT are at risk of more severe liver fibrosis (49), suggesting a bidirectional pathophysiological cross-talk between the heart and the liver. Trials with GLP-1RAs and sodium-glucose co-transporter 2 (SGLT2) inhibitors have reported reductions in EAT thickness, liver fat, and abdominal fat, often without significant BMI changes or correlation (50–55), highlighting the interrelation of these fat depots as well as their modifiable potential independent of body weight, hence the value of their quantification for risk prevention and treatment monitoring.

### 4.4 Epicardial adipose tissue and CMR markers of cardiac remodeling

Of the fat depots we assessed, a greater EAT volume was associated with adverse cardiac remodeling: expanded myocyte and interstitial volumes, increased LV mass, a more concentric LV, and higher global myocardial wall stress. The anatomic proximity of EAT to the myocardium facilitates infiltration of lipids, vasocrine and paracrine factors into the myocardium (56), rendering it susceptible to a cascade of metabolic, inflammatory, and immune activity alterations. It has been reported that paracrine exertion of EAT-derived adipokines was associated with LV systolic and diastolic functions (57). Such paracrine effects could have modulated the structural LV remodeling observed with increased EAT in the present cohort of relatively well individuals prior to discernable functional deterioration.

In insulin resistance, glucose utilization and lipolysis are reduced in EAT. Such metabolic remodeling along with the hemodynamic changes due to mechanical impediment imposed by an excessive fat pad could increase the cardiac output and energy demands of the heart, leading to elevated myocardial wall stress. The left and right filling pressures are elevated with greater EAT volume in patients with obesity and concomitant heart failure with preserved ejection fraction (HFpEF) (58). To compensate for pressure overload, cardiomyocytes undergo hypertrophic growth, manifested as increased myocyte volume and LV mass, to reduce wall stress. The disproportionate growth of wall thickness was accompanied by increased wall stress and concentricity, reflected in lower RI and mass/volume ratio, respectively. One study reported that even in patients with no HF or other cardiovascular risk factors, increased visceral adiposity was associated with myocardial steatosis, impaired myocardial energetics, increased LV mass, concentric remodeling, and diastolic dysfunction (59).

We have previously determined that increased myocardial wall stress was associated with myocardial fibrosis, possibly mediated by inflammation and immune activation (18, 60). Considering the implications of inflammation on EAT (61), we observed that a greater EAT volume was associated with an expansion of interstitial volume, a marker of diffuse interstitial myocardial fibrosis. While diabetic patients had worse cardiac remodeling (60), the present study demonstrated that the association between features of adverse cardiac remodeling and EAT amount appeared to be independent of T2DM status, hinting at a complex interplay between glucose metabolism, epicardial adiposity, and cardiac remodeling, which calls for further investigations.

### 4.5 Strengths and limitations

A strength of this study lies in the multiparametric MRI quantification of fat depots in the abdomen, liver, and heart that are metabolically important in the regulation of cardiometabolic health, providing insights into the implications of fat distribution for myocardial remodeling. Along with imaging data, comprehensive anthropometric indices offered a surrogate indication of fat composition for easy and quick measurement. Our cohort predominantly consisted of Chinese participants. Investigations on ethnic differences in fat composition, anthropometry, and implications in cardiac remodeling are warranted in future studies.

The quantification of PDFF did not account for T1 and T2* and thus estimation of liver fat may be affected by the presence or extent of inflammation, fibrosis, and iron. However, calculating PDFF using this approach was shown to provide excellent diagnostic accuracy for liver fat similar to biopsy-confirmed non-alcoholic steatohepatitis (11). Furthermore, assessing PDFF using the *LMS IDEAL* approach has been validated against lab-analyzed fat samples (15). The *LMS IDEAL* method is also robust against the fat/water swapping error. These factors increased the confidence of our PDFF analyses while acknowledging that correction for T1 and T2* should be considered for future studies involving fibrotic and inflammatory liver pathologies.

## 5 Conclusion

In Asian adults with cardiometabolic risk factors, our study, using multiparametric MRI, demonstrated that abdominal VAT and SAT have differential associations with anthropometric indices and ectopic fats. Distinctive abdominal visceral adiposity indicated by an increased VAT/SAT ratio was more prominently implicated in T2DM and was associated with increased liver and epicardial fat, the latter of which was uniquely associated with adverse cardiac remodeling. With the current data suggesting a close link between epicardial, liver, and abdominal visceral adiposity, further investigations are needed to validate the utility and reliability of measuring these fat depots using anthropometric measures such as WHR for assessment and monitoring of cardiometabolic health and remodeling.

## Data Availability

The original contributions presented in the study are included in the article/**Supplementary Material**, further inquiries can be directed to the corresponding author.
