# Supplementary material for: Differential association of abdominal, liver, and epicardial adiposity with anthropometry, diabetes, and cardiac remodeling in Asians
Source: Front Endocrinol (Lausanne). 2024 Aug 23;15:1439691. doi: 10.3389/fendo.2024.1439691 (PMC11385302; doi:10.3389/fendo.2024.1439691)
Supplement: Supplementary file 1 [file DataSheet1.pdf]

Supplementary Material

Differential Association of Abdominal, Liver and Epicardial Adiposity with Anthropometry, Diabetes and Cardiac Remodeling in Asians

Vivian Lee[1], Yiying Han[2], Desiree-Faye Toh[1], Jennifer A Bryant[2], Redha Boubertakh[1,3], Thu-Thao Le[1,3†], Calvin WL Chin[1,2,3†*]

[1]National Heart Research Institute Singapore (NHRIS), National Heart Centre Singapore, Singapore

[2]Department of Cardiology, National Heart Centre Singapore, Singapore

[3]Cardiovascular Academic Clinical Program (ACP), Duke-NUS Medical School, Singapore

[†]These authors contributed equally to this work and share senior authorship

***Correspondence:**
Calvin Chin, MD PhD FESC FRCP
Senior Consultant and Clinician Scientist, NHCS
Director of Cardiovascular Magnetic Resonance, NHCS
Associate Professor, Cardiovascular ACP, Duke-NUS Medical School
Email: cchin03m@gmail.com
Phone: (65) 6704 8905
Fax: (65) 6844 9030

Table S1. Association between abdominal adipose tissue and clinical determinants.

	**VAT, cm^2**	**SAT, cm^2**	**VAT/SAT ratio**
**Male sex**	0.29, P=0.001	−0.35, P<0.001	0.56, P<0.001
**Age, years**	0.32, P<0.001	−0.11, P=0.040	0.35, P<0.001
**Height, m**	−0.44, P<0.001	−0.39, P<0.001	−0.18, P=0.095
**Weight, kg**	0.78, P<0.001	1.03, P<0.001	−0.02, P=0.770
**SBP, mmHg**	−0.03, P=0.607	−0.02, P=0.651	−0.07, P=0.243
**Hyperlipidemia**	0.02, P=0.732	−0.04, P=0.389	0.01, P=0.935
**T2DM**	0.20, P=0.001	−0.16, P=0.002	0.27, P<0.001

Data presented as standardized β coefficients and corresponding P values analyzed with multivariable linear regression. **Abbreviations:** SAT, subcutaneous adipose tissue; SBP, systolic blood pressure; T2DM, type 2 diabetes mellitus; VAT, visceral adipose tissue.

Table S2. Differences in adiposity stratified by waist-hip ratio.

	**Normal WHR (n=38)**	**Abnormal WHR (n=110)**	**Mean difference**	**95% CI**	**P value**
**VAT, cm^2**	117.49±10.90	189.92±6.26	72.44	46.77-98.10	<0.001
**SAT, cm^2**	129.09±13.27	198.9+1±7.62	69.82	38.56-101.06	<0.001
**VAT/SAT ratio**	0.94±0.08	1.16±0.05	0.22	0.03-0.42	0.026
**EAT, cm^3**	91.35±5.75	120.72±3.30	29.37	15.84-42.89	<0.001
**Liver PDFF, %**	4.57±1.23	11.06±0.70	6.49	3.60-9.37	<0.001

ANCOVA analyses adjusted for age, sex, ethnicity, systolic blood pressure, hyperlipidemia and type 2 diabetes mellitus. Data presented as adjusted means ± standard error. Normal WHR thresholds: males <0.90; females <0.85. **Abbreviations:** CI, confidence interval; EAT, epicardial adipose tissue; PDFF, proton density fat fraction; SAT, subcutaneous adipose tissue; VAT, visceral adipose tissue; WHR, waist-hip ratio.

Table S3. Differences in adiposity stratified by BMI categories.

	Normal (n=33)	Overweight (n=86)	Obese (n=30)	P value
**VAT, cm^2**	103.96±9.60	168.23±5.70	251.71±9.68	<0.001[a,b,c]
**SAT, cm^2**	107.15±10.81	171.95±6.42	285.72±10.90	<0.001[a,b,c]
**VAT/SAT ratio**	1.02±0.09	1.13±0.05	1.10±0.09	0.599
**EAT, cm^3**	90.75±5.92	112.40±3.52	140.24±5.97	<0.001[a,b,c]
**Liver PDFF, %**	4.94±1.33	9.96±0.79	12.36±1.34	0.001[a,b]

ANCOVA analyses adjusted for age, sex, ethnicity, systolic blood pressure, hyperlipidemia and type 2 diabetes mellitus. Data presented as adjusted means ± standard error. Normal: <23 kg/m^2; overweight: 23-30 kg/m^2, obese: >30 kg/m^2. **Abbreviations:** BMI, body mass index; EAT, epicardial adipose tissue; PDFF, proton density fat fraction; SAT, subcutaneous adipose tissue; VAT, visceral adipose tissue.

[a]ANOVA *post hoc* Bonferroni adjustment: P<0.05 between normal and obese.
[b]ANOVA *post hoc* Bonferroni adjustment: P<0.05 between normal and overweight.
[c]ANOVA *post hoc* Bonferroni adjustment: P<0.05 between overweight and obese.

Table S4. Comparison of compartmental fat between individuals with and without T2DM.

	T2DM (n=56)	Non-DM (n=93)	Mean difference	95% CI	P value
**VAT, cm^2**	192.99±7.24	157.44±5.43	35.54	16.58 to 54.51	<0.001
**SAT, cm^2**	164.31±6.99	190.25±5.25	−25.94	−44.27 to −7.64	0.006
**VAT/SAT ratio**	1.33±0.07	0.96±0.05	0.37	0.18-0.56	<0.001
**EAT volume, cm^3**	124.47±4.47	106.43±3.36	18.04	6.33-29.76	0.003
**Liver PDFF, %**	11.71±1.05	7.90±0.79	3.81	1.06-6.57	0.007

ANCOVA analyses adjusted for age, sex, ethnicity, systolic blood pressure, BMI and hyperlipidemia. Data presented as adjusted means ± standard error. **Abbreviations:** BMI, body mass index; DM, diabetes mellitus; EAT, epicardial adipose tissue; PDFF, proton density fat fraction; SAT, subcutaneous adipose tissue; T2DM, type 2 diabetes mellitus; VAT, visceral adipose tissue.

Table S5. Comparison of area under the receiver operating characteristic curves between VAT/SAT, VAT and SAT for the discrimination of T2DM status.

	**z statistic**	**AUC difference**	**Standard error**	**95% CI**	**P value**
**VAT/SAT − VAT**	2.174	0.075	0.280	0.007-0.143	0.030
**VAT/SAT − SAT**	3.847	0.175	0.286	0.086-0.263	<0.001

AUC of VAT/SAT, VAT and SAT, respectively: 0.789, 0.714, 0.615. **Abbreviations:** AUC, area under the curve; CI, confidence interval; SAT, subcutaneous adipose tissue; VAT, visceral adipose tissue.

Table S6. Associations between fat depots and cardiac remodeling features.

	**Indexed LV mass, g/m^2**	**Indexed interstitial volume, mL/m^2**	**Indexed myocyte volume, mL/m^2**	**LV mass/EDV ratio**	**Remodeling index**
**EAT volume, cm^3**	0.24, P=0.004	0.19, P=0.023	0.26, P=0.001	0.18, P=0.035	−0.18, P=0.023
**VAT/SAT ratio**	0.08, P=0.436	0.07, P=0.541	0.06, P=0.578	0.23, P=0.035	−0.13, P=0.189
**Liver PDFF, %**	−0.08, P=0.346	−0.15, P=0.069	−0.05, P=0.575	0.10, P=0.225	−0.15, P=0.054

Multivariable linear regression adjusted for age, sex, ethnicity, systolic blood pressure, hyperlipidemia and type 2 diabetes mellitus. BMI was not included for adjustment because cardiac parameters were indexed to body surface area, where appropriate. Data presented as standardized β coefficients and corresponding P values. **Abbreviations:** EAT, epicardial adipose tissue; EDV, end-diastolic volume; LV, left ventricular; PDFF, proton density fat fraction; SAT, subcutaneous adipose tissue; VAT, visceral adipose tissue.

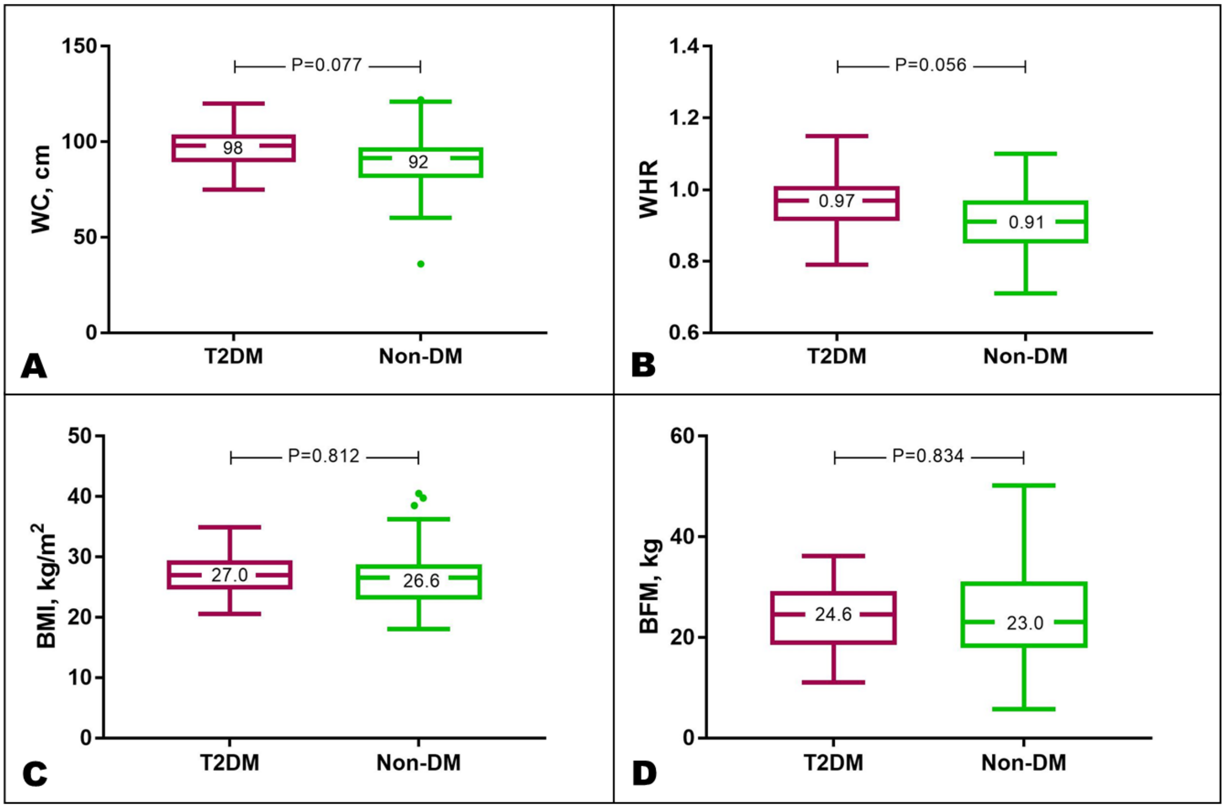

Figure S1. Anthropometric measures of adiposity between individuals with and without T2DM.

ANCOVA analyses adjusted for age, sex, ethnicity, systolic blood pressure and hyperlipidemia. Results presented as Tukey box and whisker plots; P values are from adjusted analyses. **Abbreviations:** BFM, body fat mass on bioimpedance; BMI, body mass index; DM, diabetes mellitus; T2DM, type 2 diabetes mellitus; WC, waist circumference; WHR, waist-hip ratio.
